# Therapeutic Potential of *Salvia miltiorrhiza* Root Extract in Alleviating Cold-Induced Immunosuppression

**DOI:** 10.3390/ijms25179432

**Published:** 2024-08-30

**Authors:** Chi-Cheng Li, Song-Lin Liu, Te-Sheng Lien, Der-Shan Sun, Ching-Feng Cheng, Hussana Hamid, Hao-Ping Chen, Tsung-Jung Ho, I-Hsin Lin, Wen-Sheng Wu, Chi-Tan Hu, Kuo-Wang Tsai, Hsin-Hou Chang

**Affiliations:** 1 Department of Hematology and Oncology, Buddhist Tzu Chi General Hospital, Hualien 970, Taiwan; kevinlcc1234@gmail.com; 2 Center of Stem Cell & Precision Medicine, Hualien Tzu Chi Hospital, Hualien 970, Taiwan; 3 Department of Molecular Biology and Human Genetics, Tzu Chi University, Hualien 970, Taiwan; archer830616@gmail.com (S.-L.L.); alan211@mail.tcu.edu.tw (T.-S.L.); dssun@mail.tcu.edu.tw (D.-S.S.); 4 Department of Pediatrics, Taipei Tzu Chi Hospital, Buddhist Tzu Chi Medical Foundation, New Taipei City 231, Taiwan; chengcf@mail.tcu.edu.tw; 5 Institute of Biomedical Sciences, Academia Sinica, Taipei 115, Taiwan; 6 Department of Biochemistry, School of Medicine, Tzu Chi University, Hualien 970, Taiwan; hamidhussana@gmail.com (H.H.);; 7 Integration Center of Traditional Chinese and Modern Medicine, Hualien Tzu Chi Hospital, Hualien 970, Taiwan; tjho@tzuchi.com.tw; 8 Department of Chinese Medicine, Hualien Tzu Chi Hospital, Hualien 970, Taiwan; 9 School of Post-Baccalaureate Chinese Medicine, Tzu Chi University, Hualien 970, Taiwan; ccmp220@mail.tcu.edu.tw; 10 Division of General Surgery, Department of Surgery, Hualien Tzu Chi Hospital, Buddhist Tzu Chi Medical Foundation Hualien, Hualien 970, Taiwan; wuws@gms.tcu.edu.tw; 11 Department of Laboratory Medicine and Biotechnology, College of Medicine, Tzu Chi University Hualien, Hualien 970, Taiwan; 12 Research Center for Hepatology, Department of Gastroenterology, Buddhist Tzu Chi General Hospital, Buddhist Tzu Chi Medical Foundation, Hualien 970, Taiwan; chitan.hu@msa.hinet.net; 13 Department of Research, Taipei Tzu Chi Hospital, Buddhist Tzu Chi Medical Foundation, New Taipei City 231, Taiwan; tch33225@tzuchi.com.tw

**Keywords:** ambient cold exposure, immunomodulation, red sage, Danshan, tanshinone IIA, mouse model, circulating immunoglobulin, bacterial clearance

## Abstract

The interaction between environmental stressors, such as cold exposure, and immune function significantly impacts human health. Research on effective therapeutic strategies to combat cold-induced immunosuppression is limited, despite its importance. In this study, we aim to investigate whether traditional herbal medicine can counteract cold-induced immunosuppression. We previously demonstrated that cold exposure elevated immunoglobulin G (IgG) levels in mice, similar to the effects of intravenous immunoglobulin (IVIg) treatments. This cold-induced rise in circulating IgG was mediated by the renin–angiotensin–aldosterone system and linked to vascular constriction. In our mouse model, the cold-exposed groups (4 °C) showed significantly elevated plasma IgG levels and reduced bacterial clearance compared with the control groups maintained at room temperature (25 °C), both indicative of immunosuppression. Using this model, with 234 mice divided into groups of 6, we investigated the potential of tanshinone IIA, an active compound in *Salvia miltiorrhiza* ethanolic root extract (SMERE), in alleviating cold-induced immunosuppression. Tanshinone IIA and SMERE treatments effectively normalized elevated plasma IgG levels and significantly improved bacterial clearance impaired by cold exposure compared with control groups injected with a vehicle control, dimethyl sulfoxide. Notably, bacterial clearance, which was impaired by cold exposure, showed an approximately 50% improvement following treatment, restoring immune function to levels comparable to those observed under normal temperature conditions (25 °C, *p* < 0.05). These findings highlight the therapeutic potential of traditional herbal medicine in counteracting cold-induced immune dysregulation, offering valuable insights for future strategies aimed at modulating immune function in cold environments. Further research could focus on isolating tanshinone IIA and compounds present in SMERE to evaluate their specific roles in mitigating cold-induced immunosuppression.

## 1. Introduction

The interplay between environmental stressors and immune function is a critical area of research with significant implications for human health. Among these stressors, ambient cold exposure has been recognized as a potent modulator of immune responses, often leading to a state of immunosuppression [1,2,3,4,5,6,7,8]. Immunosuppressed individuals face significant health challenges, given that their weakened immune systems cause them to become more vulnerable to infections and other illnesses [9,10,11,12]. This increased susceptibility can lead to more severe disease progression, longer recovery times, and complications such as chronic inflammation or organ damage [9]. Immunosuppression can arise from various factors, including chronic diseases and medical treatments, such as chemotherapy, as well as the prolonged use of immunosuppressive medications [13,14,15,16,17]. In addition, cold exposure has been shown to exacerbate immunosuppression, potentially leading to further immune dysfunction [1,2,3,4,5,6,7,8]. Understanding the interplay between immunosuppression and cold exposure is crucial to developing strategies to protect and improve the health of these vulnerable individuals.

These observations align with the findings of historical physicians, such as Hippocrates (460–370 BC) and Zhang Zhongjing (150–219 AD), who noted the connection between cold exposure and various illnesses [18,19]. This phenomenon, marked by a reduction in the immune system’s ability to fight infections and diseases, supports the observation that low ambient temperatures correlate with seasonal patterns in the prevalence of respiratory infections and influenza virus outbreaks [20,21,22,23,24,25,26,27]. However, despite long-standing recognition, the considerable impact of cold-induced immunosuppression and the pressing need for specific therapeutic strategies, especially in light of recent extreme weather conditions, remain inadequately addressed, with limited research on the molecular mechanisms and novel therapeutic approaches.

We previously demonstrated that cold exposure elevates circulating immunoglobulin G (IgG) levels in mice, inducing a state of immunosuppression similar to that achieved by intravenous immunoglobulin (IVIg) treatments for inflammatory diseases [1]. IVIg, a purified immunoglobulin fraction derived from the plasma of healthy donors, is commonly used in clinical settings to treat various autoimmune and systemic inflammatory disorders [28,29]. Its mechanisms are complex, involving the modulation of Fc receptors, interference with complement activation, and effects on the activation and differentiation of leukocyte subsets [28,29,30,31]. Cold-induced IgG elevation and subsequent immunosuppression are mediated through the activation of the renin–angiotensin–aldosterone (RAA) system via transient receptor potential melastatin 8 (TRPM8), leading to hypertension and impaired bacterial clearance in vivo [1]. This model facilitates the monitoring of cold-induced immunosuppression and its reversal by assessing plasma IgG levels and bacterial clearance in mice.

Danshan, a medicinal preparation from the roots of red sage (*Salvia miltiorrhiza* Bunge), is available in water- and ethanol-extract forms and has been traditionally used in Chinese medicine for its well-established anti-inflammatory and immunomodulatory properties [32]. Tanshinone IIA, an active compound of Danshan, has shown potential in modulating immune responses disrupted by cold exposure through its effects on cellular pathways involved in immune regulation [32,33] and its antihypertensive effects in vivo [34,35,36]. Since cold-induced hypertension can increase plasma IgG levels, and the antihypertensive drug losartan has been shown to reduce cold-induced immunosuppression [1], tanshinone IIA, with its antihypertensive effects, might also ameliorate cold-induced immunosuppression. To explore this possibility, we conducted the present study.

## 2. Results

### 2.1. Cold-Induced Immunosuppression Revealed by Ameliorated Immune Thrombocytopenia and Increased Circulating IgG Levels in Mice

**Figure 1:**
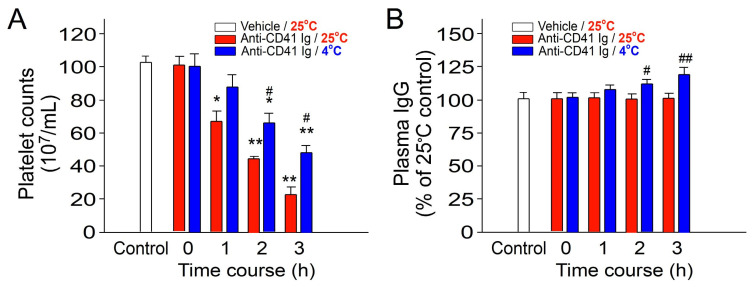
Treatment involving cold exposure resulted in decreased immune thrombocytopenia (ITP) and increased plasma IgG levels in mice. (**A**) Platelet counts and (**B**) plasma total immunoglobulin G (IgG) levels were assessed in ITP mice injected with anti-CD41 antibody at 0–3 h post induction of ITP. All data are expressed as the mean ± standard error. The results represent three independent experiments with two mice per group (*n* = 6). * *p* < 0.05 and ** *p* < 0.01 compared with the 0 h groups; ^#^ *p* < 0.05 and ^##^ *p* < 0.01 compared with the respective 25 °C groups. The average plasma IgG level in the control groups was normalized as 100% (B).

Following established procedures, we replicated the experiment examining the cold-induced suppression of immune thrombocytopenia (ITP) (Figure 1). We observed a kinetic decline in platelet counts, indicative of ITP, in mice treated with anti-CD41 Ig (CD41 being a platelet surface marker). This ITP phenomenon was mitigated by cold exposure, confirming immunosuppression (Figure 1A, platelet counts). Furthermore, cold exposure led to a significant increase in plasma IgG levels in mice (Figure 1B), a phenomenon akin to IVIg-induced immunosuppression.

### 2.2. Reversal of Cold-Induced Immunosuppression in Mice through Treatment with a S. miltiorrhiza Root Component Tanshinone IIA

**Figure 2:**
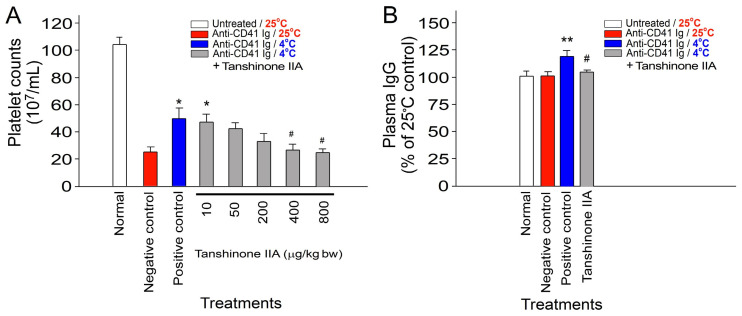
Treatment involving tanshinone IIA reversed the reduction in immune thrombocytopenia (ITP) caused by cold exposure and mitigated the cold-induced elevation of plasma IgG levels in mice. (**A**) Platelet counts and (**B**) plasma total immunoglobulin G (IgG) levels were evaluated in ITP mice injected with an anti-CD41 antibody at 0–3 h post ITP induction, with or without additional intraperitoneal administration of tanshinone IIA (10–800 μg/kg bw in (**A**), 800 μg/kg bw in (**B**)), administered concurrently with cold exposure. The control groups, which included the normal, negative control (without immunosuppression), and positive control (with immunosuppression) groups, were treated with DMSO only at different temperatures. DMSO served as the solvent to dissolve tanshinone IIA. All data are presented as the mean ± standard error. The findings represent three independent experiments with two mice per group (*n* = 6). * *p* < 0.05 and ** *p* < 0.01 compared with the negative control groups; ^#^ *p* < 0.05 compared with the positive control groups. The average plasma IgG level in the untreated groups was normalized to 100% (**B**).

Mice were intraperitoneally administered varying doses of tanshinone IIA, ranging from 10 to 800 μg/kg body weight, to investigate the potential protective effects of tanshinone IIA against cold-induced immunosuppression. Our results demonstrate that treatment with tanshinone IIA at doses between 400 and 800 μg/kg body weight effectively ameliorated cold-induced immunosuppression in the ITP experiment. Furthermore, administration of tanshinone IIA at a dosage of 800 μg/kg body weight markedly reduced the cold-induced elevation of plasma IgG levels in mice. This result indicates the amelioration of cold-induced IVIg-like immunosuppression (Figure 2).

**Figure 3:**
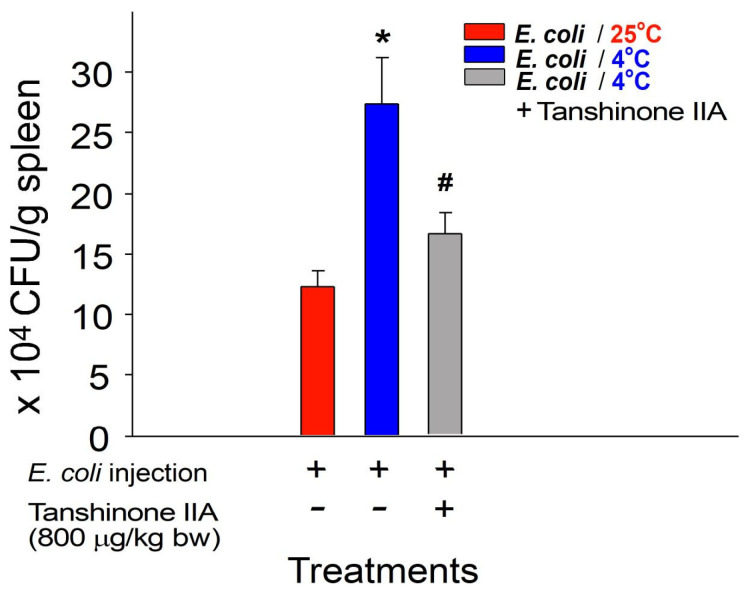
Treatment involving tanshinone IIA reversed the suppression of bacterial clearance induced by cold exposure in mice. The viable bacterial counts (colony-forming unit, CFU) of *Escherichia coli* (BL21, 6 × 10^9^ CFU/kg)-injected mice were assessed 24 h after cold exposure; surviving bacteria (CFU) were quantified from spleen tissues of mice with or without tanshinone IIA (800 μg/kg) treatments. The vehicle control (tanshinone IIA−) groups were treated with DMSO, which was used as a solvent to dissolve tanshinone IIA. All data in this figure are expressed as the mean ± standard error. The results represent three independent experiments with two mice per group (*n* = 6). * *p* < 0.05 compared with the 25 °C groups; ^#^ *p* < 0.05 compared with the 4 °C vehicle control groups without tanshinone IIA treatment.

To further investigate the potential protective effects of tanshinone IIA against the cold-induced suppression of bacterial clearance in vivo, bacterial clearance was assessed by homogenizing mouse spleens in phosphate-buffered saline (PBS) and then quantifying the surviving bacteria (colony-forming unit (CFU)) using the standard plating method [37,38]. Experimental mice were administered tanshinone IIA intraperitoneally at a dosage of 800 μg/kg body weight. This dosage was selected based on its effectiveness in reversing the cold-induced effects observed in the ITP experiment (Figure 2A). Our findings demonstrate that tanshinone IIA treatment at this dosage effectively ameliorated the cold-induced suppression of bacterial clearance in mice (Figure 3: viable bacteria in the mouse spleen). These results suggest that tanshinone IIA, as an active component of *S. miltiorrhiza* ethanolic root extract (SMERE), is effective in alleviating cold-induced immunosuppression.

### 2.3. Reversal of Cold-Induced Immunosuppression through Treatment with SMERE in Mice

**Figure 4:**
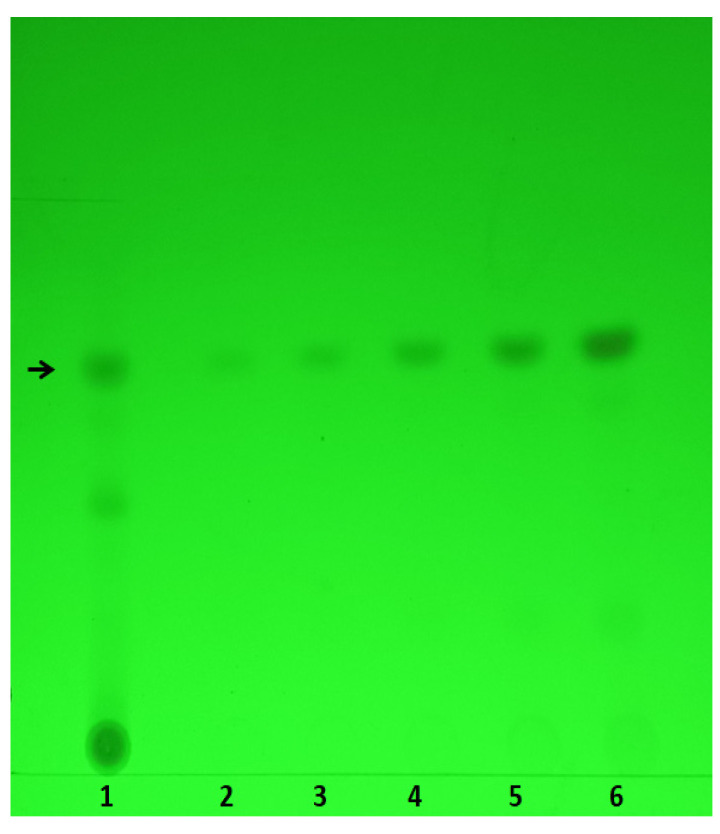
Results of thin-layer chromatography analysis. The black arrow indicates the position of tanshinone IIA. Lane 1 represents crude sample of the *S. miltiorrhiza* root ethanolic extract (SMERE), whereas lanes 2 to 6 refer to tanshinone IIA standards (ranging from 6.25 μg to 100 μg; Tocris Bioscience, high performance liquid chromatography (HPLC)-grade). The TLC plate was visualized under ultraviolet (UV) light, and band intensity was measured using ImageJ software (version 1.52a). The content of tanshinone IIA was calculated via comparison with the standards. The data indicate that the tanshinone IIA content in the SMERE sample used for this study is 18.6% (*w*/*w*).

**Figure 5:**
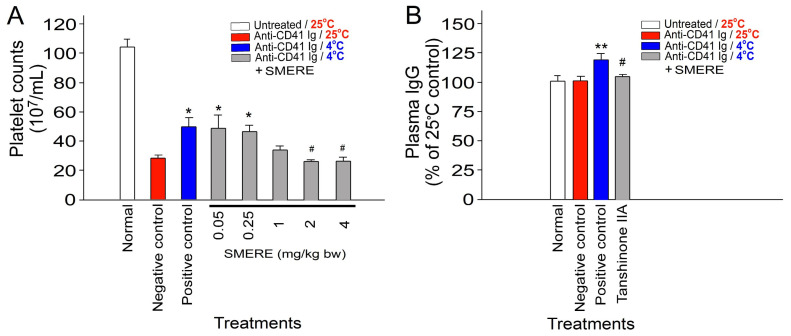
Treatment involving SMERE reversed the reduction in immune thrombocytopenia (ITP) caused by cold exposure and mitigated the cold-induced elevation of plasma IgG levels in mice. (**A**) Platelet counts and (**B**) plasma total immunoglobulin G (IgG) levels were evaluated in ITP mice injected with an anti-CD41 antibody at 0–3 h post ITP induction, with or without additional intraperitoneal administration of SMERE (0.05–4 mg/kg), administered concurrently with cold exposure. The control groups, which included the normal, negative control (without immunosuppression), and positive control (with immunosuppression) groups, were treated with DMSO only at different temperatures. DMSO served as the solvent for dissolving SMERE. All data are presented as the mean ± standard error. The findings represent three independent experiments with two mice per group (*n* = 6). * *p* < 0.05 and ** *p* < 0.01 compared with the negative control groups; ^#^ *p* < 0.05 compared with the positive control groups. The average plasma IgG level in the untreated groups was normalized to 100% (**B**).

We found that the SMERE in our preparation contained up to 18% tanshinone IIA through thin-layer chromatography (TLC) analysis (Figure 4). To further explore the potential protective role of SMERE against cold-induced immunosuppression, mice were intraperitoneally administered various doses of SMERE, ranging from 0.05 to 4 mg/kg body weight. In agreement with tanshinone IIA experiments, our findings reveal that treatment with SMERE at doses of 2–4 mg/kg body weight effectively attenuated cold-induced immunosuppression in ITP. In addition, treatment with 4 mg/kg body weight of SMERE significantly mitigated the cold-induced elevation of plasma IgG levels in mice, indicating the alleviation of cold-induced IVIg-like immunosuppression in mice (Figure 5).

**Figure 6:**
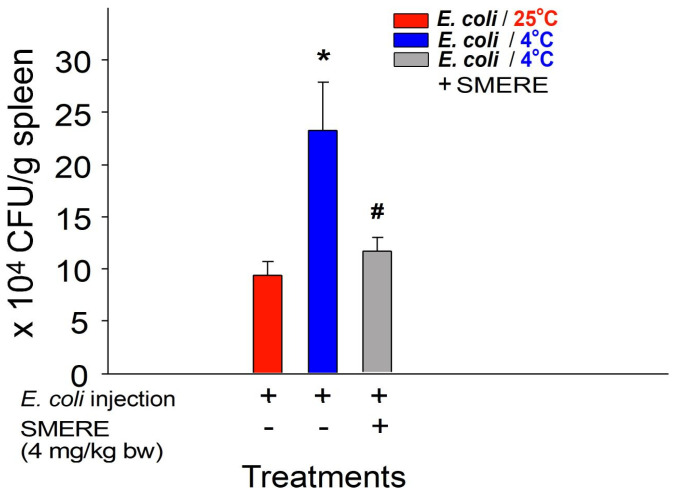
Treatment involving SMERE reversed the suppression of bacterial clearance induced by cold exposure in mice. The viable bacterial counts (CFU) of *E. coli* (BL21, 6 × 10^9^ CFU/kg)-injected mice were assessed 24 h after cold exposure; surviving bacteria (CFU) were quantified from spleen tissues of mice with or without SMERE treatments (4 mg/kg). The vehicle control groups were treated with DMSO only, which was used as a solvent to dissolve SMERE. All data in this figure are expressed as the mean ± standard error. The results represent three independent experiments with two mice per group (*n* = 6). * *p* < 0.05 compared with the 25 °C groups, ^#^ *p* < 0.05 compared with the 4 °C vehicle control groups without SMERE treatment.

Mice were intraperitoneally administered the SMERE at a dosage of 4 mg/kg body weight to further investigate the potential protective effects of SMERE against cold-induced suppression of bacterial clearance in vivo. In agreement with tanshinone IIA experiments, our findings indicate that administering SMERE at this dose effectively alleviated the cold-induced inhibition of bacterial clearance in mice, as evidenced by enhanced bacterial survival in the mouse spleen (Figure 6, viable bacteria in the mouse spleen). Again, these results suggest that treatments with SMERE alleviated cold-induced immunosuppression in mice.

## 3. Discussion

In bacterial clearance experiments, consistent with previous findings [1,8,39,40], we observed that ambient cold exposure significantly suppressed bacterial clearance in vivo. However, treatment with tanshinone IIA and SMERE notably ameliorated cold-induced suppression of bacterial clearance in experimental animals (Figure 3 and Figure 6). These findings highlight the therapeutic potential of tanshinone IIA and SMERE in mitigating the adverse effects of cold exposure on immune function.

The current landscape of pharmacological interventions for cold-induced immunosuppression is notably sparse, with a pressing need for research on and development of drugs targeting this specific form of immune dysregulation. Despite the recognition of cold exposure as a significant environmental stressor that can diminish immune function, therapeutic strategies to counteract this effect remain underexplored. The urgent need for such interventions is underscored by the seasonal patterns of respiratory infections and the potential for the exacerbation of chronic inflammatory conditions during colder months. Here, we found that tanshinone IIA, a compound in SMERE with antihypertensive properties, can ameliorate cold-induced immunosuppression. This finding suggests that other herbal compounds with antihypertensive effects may be candidates for mitigating cold-induced side effects and thus may warrant further investigation. Interestingly, ginger (*Zingiber officinale*) and cinnamon (*Cinnamomum cassia*) are commonly used in treatments for cold-induced effects [41,42,43,44], exhibiting antihypertensive properties [45,46,47]. Traditional Chinese herbal formulas, such as Guizhi Tang, containing cinnamon twigs and fresh ginger, and Xiao Qinglong Tang, with cinnamon twigs and dried ginger, are used to counteract cold exposure effects including allergic airway inflammation, coughing, and nasal congestion [48,49,50,51,52]. These findings suggest that these herbal drugs and formulas may theoretically partially ameliorate cold-induced adverse effects through their antihypertensive properties. Therefore, this hypothesis and the antihypertensive compounds in ginger and cinnamon warrant further exploration. In addition, other components of Danshan, such as water-soluble salvianolic acids, known for their antihypertensive and vascular modulation effects [53,54,55,56,57], are worthy of further study.

Cold-induced immunosuppression involves complex interactions among environmental stimuli, physiological responses, and immune regulation, traditionally linked to heightened cortisol and corticosterone levels [5,58]. However, recent research has revealed that wild-type and B-cell-deficient (*Ighm^−/−^*) mice exhibited cold-induced elevation of cortisol and corticosterone levels. Interestingly, only wild-type and not B-cell-deficient mice lacking circulating IgG expression showed cold-induced immunosuppression, suggesting the essential role of IgG expression rather than the elevation of cortisol and corticosterone levels in cold-induced immunosuppression in mice [1]. These results uncovered pathways contributing to cold-induced immunosuppression, particularly the elevation of circulating IgG levels reminiscent of IVIg treatments [1]. Activation of the RAA system via TRPM8 plays a crucial role, leading to hypertension, increased plasma Ig levels, and subsequent immunosuppression, including impaired bacterial clearance [1]. Moreover, vasoconstriction during cold exposure exacerbates these effects by promoting plasma leakage and further elevating IgG levels. As a result, treatment with an anti-hypertensive vessel-dilating drug losartan, an angiotensin II receptor blocker, can effectively rescue cold-induced immunosuppression in mice [1].

Danshan holds a prominent place in traditional Chinese medicine due to its confirmed anti-hypertensive and immunomodulatory effects [59,60]. Danshan and its constituent tanshinone IIA have been acknowledged for their anti-hypertensive properties, attributed to their ability to induce vasorelaxation [33,34,36,59,60,61,62]. Consequently, based on the previously discussed losartan-mediated rescue in cold-induced immunosuppression [1], treatments with tanshinone IIA and SMERE may counteract cold-induced vasoconstriction, thereby mitigating the subsequent elevation of plasma IgG levels and the resulting immunosuppression.

In addition to its vascular regulatory effects, tanshinone IIA has been extensively studied for its immunomodulatory and anti-inflammatory properties [32,33,63,64,65,66,67,68,69,70]. Research has shown that tanshinone IIA can inhibit the production of key pro-inflammatory cytokines such as tumor necrosis factor-α, interleukin (IL)-1β, and IL-6, thereby reducing inflammation [63,64,71]. It also modulates the activity of various immune cells, including macrophages and T cells, contributing to its broad anti-inflammatory effects [65,72,73,74,75]. This compound exerts these effects by modulating key signaling pathways, such as nuclear factor-κB and mitogen-activated protein kinase [75,76,77], which are crucial in the regulation of immune responses and inflammation. Furthermore, tanshinone IIA has been found to protect against oxidative stress and cell death in inflammatory settings [71,78,79], further supporting its potential as a therapeutic agent in treating inflammatory diseases. These findings suggest that tanshinone IIA could be a valuable addition to the therapeutic arsenal for managing conditions characterized by excessive inflammation and immune dysregulation. The observed ameliorative effects of tanshinone IIA on cold-induced immune dysregulation align with its established anti-inflammatory and immune-modulatory roles given that cold exposure can also lead to inflammation, cellular stress, and tissue damage [80,81,82].

We conducted a series of experiments using an ITP mouse model and an in vivo bacterial clearance assay to investigate the effects of tanshinone IIA and SMERE, validating the results regarding the alleviation of cold-induced immunosuppression. Treatment with SMERE effectively reduced cold-induced immunosuppression, as demonstrated by the reversal of cold-induced ITP and the reduction in cold-induced elevation of plasma IgG levels (Figure 2 and Figure 5).

Our findings demonstrating the efficacy of tanshinone IIA and SMERE in reversing cold-induced immunosuppression highlight the promise of traditional herbal medicine as a source for novel therapeutic agents. These results suggest the necessity for a more comprehensive study to thoroughly investigate these potential effects. Further research is imperative to translate our preclinical findings into clinical applications. Cautious extrapolation of findings to humans is required given that this study was conducted using preclinical animal models. Additional clinical studies are warranted to assess the safety, efficacy, and optimal dosing regimens of tanshinone IIA and SMERE in human populations exposed to cold-weather conditions. Moreover, while we have delineated the immunomodulatory effects of tanshinone IIA and SMERE, the underlying molecular mechanisms remain incompletely understood. Future studies should focus on elucidating the specific cellular and molecular pathways involved in mediating the protective effects of these compounds against cold-induced immunosuppression.

## 4. Materials and Methods

### 4.1. Laboratory Mice

Wild-type C57BL/6J mice, aged 8–12 weeks and weighing approximately 27 g on average, were obtained from the National Laboratory Animal Center in Taipei, Taiwan. The mice were then housed in the Animal Center of Tzu-Chi University under specific pathogen-free conditions, with controlled lighting and temperature, and were provided unrestricted access to food and filtered water. A total of 234 mice were used in the experiments.

Using the established mouse model of cold-exposure-induced immunosuppression, we aimed to explore the potential of the traditional herbal medicine SMERE and tanshinone IIA in mitigating the immunosuppressive effects induced by cold exposure. We employed two primary assay systems for this purpose following the methods described in [1]. The first approach involved inducing immune thrombocytopenia in mice using anti-CD41 Ig, where cold exposure tends to suppress this condition [1]. The second assay entailed injecting mice with *E. coli* bacteria to observe in vivo bacterial clearance in the spleen, which is typically suppressed under cold exposure conditions [1,83]. The methods related to the measurement of plasma IgG levels, the induction of experimental immune thrombocytopenia (ITP), and the analysis of bacterial clearance in the cold exposure mouse model are detailed in the following sections.

### 4.2. Measurements of Plasma IgG Levels in the Cold Exposure Mouse Model

The cold exposure mouse model, inducing an immunosuppressive state, was developed using previously reported protocols [1,83]. In this model, C57BL/6J mice were subjected to acute cold exposure in a 4 °C room, whereas the control group mice were placed in a 25 °C room, with both conditions lasting for 4–7 h prior to blood collection. In the 4 °C environment, each mouse was housed individually to prevent them from huddling together for warmth, influencing the analysis results and increasing the variations. Following blood collection, the samples were mixed with an acid–citrate–dextrose (ACD) anticoagulant solution (38 mM citric acid, 75 mM sodium citrate, and 100 mM dextrose) [30,84,85] in a blood-to-ACD ratio of 4:1 to prevent coagulation. After removing blood cells and platelets through centrifugation at 2500× *g* for 10 min (centrifuge model Z323K; Hermle LaborTechnik, Wehingen, Germany), the relative plasma IgG levels in mice were assessed using an enzyme-linked immunosorbent assay (ELISA) with plasma samples diluted 200-fold in PBS. The IgG levels of untreated 25 °C control mice were used as the baseline and normalized to 100%. The ELISA analysis employed an anti-mouse IgG secondary antibody (Jackson ImmunoResearch, West Grove, PA, USA), as detailed in a previous study [1].

### 4.3. Induction of Experimental Immune Thrombocytopenia (ITP) in the Cold Exposure Mouse Model

ITP was induced and treated as previously described [30]. Mice received an intravenous injection of 0.1 mg/kg body weight of anti-platelet monoclonal antibodies (rat anti-mouse integrin αIIb/CD41 Ig, clone MWReg30; BD Biosciences, Franklin Lakes, NJ, USA) to induce ITP. Whole-blood samples (100–120 μL) were collected from the mice in Eppendorf tubes containing ACD anticoagulant solution to assess platelet count. In the cold exposure mouse model, anti-CD41 Ig was administered 4 h post cold exposure to induce ITP to investigate the mitigating effects of ITP under cold conditions (4 °C). Platelet counts were then measured using a hematology analyzer (KX-21N, Sysmex, Kobe, Japan) [86] 3 h after ITP induction. Concurrently, treatments with tanshinone IIA and SMERE were initiated at the beginning of cold exposure.

### 4.4. Analysis of Bacterial Clearance in the Cold Exposure Mouse Model

*Escherichia coli* was cultured using conventional techniques [87]. Experimental mice were challenged with intravenous administration of bacteria (*E. coli*, BL21, 6 × 10^9^ CFU/kg, with bacterial cells collected during the log phase of growth; no mortality of mice observed within 24 h at 4 °C) to investigate the immunosuppressive effects of cold exposure on bacterial clearance. Before cold exposure treatments, a 40 h period of circulating equilibrium at 25 °C was maintained for all mice (control groups remained at 25 °C). Treatments with tanshinone IIA and SMERE were administered at the start of the cold exposure. One day after the cold exposure treatments, the mice in 25 °C and 4 °C groups were euthanized, and their spleens were harvested and weighed. The spleen tissue samples were homogenized in ice-cold PBS using a homogenizer (BioSpec Products, Racine, WI, USA) until no visible solid tissue remained. The homogenate was then filtered through a 150 µm nylon mesh to remove residual tissue debris. The surviving bacteria in the filtered supernatant were quantified using the standard plating method [37,38]. After incubation at 37 °C for 24 h, *E. coli* colonies were counted on lysogeny broth–agar plates (Sigma–Aldrich, St. Louis, MO, USA). Mouse groups showing higher levels of surviving bacteria in the spleen after cold exposure (4 °C) compared to those kept at normal temperature (25 °C) were classified as immunosuppressed.

### 4.5. Tanshinone IIA, and S. miltiorrhiza Root Ethanolic Extract

Powder of *S. miltiorrhiza* root was purchased from Sun Ten Pharmaceutical Co., Ltd. (New Taipei City, Taiwan). The SMERE was prepared using a 200 mg sample of crude powder dissolved in 10 mL of 95% ethanol and incubated at 25 °C for 2 h. After centrifuging at 10,000× *g* for 10 min at 25 °C (centrifuge model Z323K; Hermle LaborTechnik), the solution was filtered through a 0.22 μm polyvinylidene difluoride syringe filter (Thermo Fisher Scientific, Waltham, MA, USA) to remove undissolved debris. Thin-layer chromatography (TLC) analysis was performed on a Silica Gel 60 F254 TLC plate (Merck KGaA, Darmstadt, Germany) to determine the tanshinone IIA content in the *S. miltiorrhiza* root sample. The solvent used for TLC was a mixture of ethyl acetate, toluene, and methanol at a 10:30:1 ratio. The TLC plate was visualized under UV light, and band intensity was measured using ImageJ software (version 1.52a, NIH, Bethesda, MD, USA). The tanshinone IIA content was calculated by comparing it to a set of standards (Figure 4). Tanshinone IIA, with a purity of 98%, was procured from Tocris Bioscience (Minneapolis, MN, USA) as HPLC-grade. The tanshinone IIA content was determined via comparison with standards, revealing that the SMERE sample used in this study contained 18.6% (*w*/*w*) tanshinone IIA, which corresponds to a yield of 186 mg/g of SMERE. In the mouse models, tanshinone IIA and SMERE were dissolved in DMSO and administered intraperitoneally at various doses, as detailed in the corresponding results sections and figure legends. Specifically, tanshinone IIA was prepared at a concentration of 1 mg/mL and SMERE at 4 mg/mL. On the basis of these concentrations, 20 and 25 μL DMSO injections per mouse were required for the vehicle control groups to administer 800 μg/kg of tanshinone IIA and 4 mg/kg of SMERE to 25 g mice, respectively.

### 4.6. Statistical Analyses

The experimental data were analyzed using Microsoft Office Excel 2003 and SPSS 17. The data were presented as the mean ± standard error of replicates. Statistical significance was assessed using a one-way analysis of variance followed by post hoc Bonferroni-corrected *t*-tests. A significance level of α = 0.05 was used, indicating the threshold for statistical significance.

## 5. Conclusions

In conclusion, our study demonstrates the therapeutic potential of tanshinone IIA, a key component of SMERE, in mitigating cold-induced immunosuppression by restoring plasma IgG levels and enhancing bacterial clearance in mouse models. These findings highlight the immunomodulatory properties of both tanshinone IIA and SMERE, suggesting their promise in developing new interventions to strengthen immune function during cold exposure. Future research should focus on isolating tanshinone IIA and other compounds in SMERE to evaluate their specific roles in counteracting cold-induced immunosuppression, as well as conducting clinical studies to confirm their efficacy and safety in humans. Additionally, mechanistic studies are needed to clarify the cellular and molecular pathways through which these compounds exert their immunomodulatory effects.

## Data Availability

The datasets generated and analyzed during this study are available from the corresponding author upon reasonable request.
